# Association of *HOMER1* rs2290639 with suicide attempts in Hong Kong Chinese and the potentially functional role of this polymorphism

**DOI:** 10.1186/s40064-016-2404-1

**Published:** 2016-06-17

**Authors:** Shitao Rao, Marco H. B. Lam, Venus S. Y. Yeung, Yun Kwok Wing, Mary Miu Yee Waye

**Affiliations:** Croucher Laboratory for Human Genomics, Rm324A, Lo Kwee-Seong Integrated Biomedical Sciences Building, School of Biomedical Sciences, Area 39; The Nethersole School of Nursing, The Chinese University of Hong Kong, Shatin, N.T. Hong Kong; Department of Psychiatry, Shatin Hospital, The Chinese University of Hong Kong, 33 Ah Kong Kok Street, Shatin, N.T. Hong Kong

**Keywords:** Suicidal attempts, Major depression disorder, *HOMER1* rs2290639, Meta-analysis, Functional role, Psychometric properties

## Abstract

**Objective:**

Animal evidence and genetic studies suggest that *HOMER1* (homer homolog 1) is involved in the etiology of suicidal behavior and major depression disorder (MDD). However, most of genetic studies were performed in Caucasians and the potentially functional role of associated polymorphisms in *HOMER1* was seldom reported. The purpose of this study was to investigate the association of a *HOMER1* polymorphism rs2290639 with suicide attempts (SA) and MDD in Hong Kong Chinese, and then briefly elucidate the potentially functional role of the associated polymorphism.

**Methods:**

NEO personality inventory, impulsiveness and depression rating scales were completed by the subjects. The association studies of *HOMER1* rs2290639 with SA or MDD were performed by case–control association studies. The bioinformatics analyses were adapted to predict potential transcription factors binding sites for the associated polymorphism.

**Results:**

The association studies and meta-analysis suggested that the *HOMER1* rs2290639 was significantly associated with susceptibility to SA but seemed not to be associated with MDD in Hong Kong Chinese. This polymorphism might affect the transcription of the *HOMER1* gene through interacting with a reliable transcription factor as found by three of four bioinformatics tools. In addition, close correlations between impulsiveness and NEO personality five factors were found in SA and MDD patients, which provide a possible way to assess the impulsiveness of patients through subjects’ personality profiles for Hong Kong Chinese.

**Conclusions:**

The *HOMER1* rs2290639 polymorphism was significantly associated with susceptibility to SA in Hong Kong Chinese affected by psychiatric disorders, which might be explained by the potentially functional role of this polymorphism.

**Electronic supplementary material:**

The online version of this article (doi:10.1186/s40064-016-2404-1) contains supplementary material, which is available to authorized users.

## Background

Approximately 1 million people worldwide die by suicide each year, accounting for 1.5 % of death by all causes (Mann 2003). Completed suicide is the 10th leading cause of death worldwide and suicide attempts (i.e. non-fatal suicidal behavior) is up to 20 times more frequent than completed suicide (Hawton and van Heeringen 2009; Varnik 2012), which confirms that suicidal behavior means a heavy burden on the health-care system and alerts the severity of its corrosive social impact (Miller et al. 2012b).

Suicidal behavior is generally regarded as a complex health and social issue that is believed to manifest as a combination of many factors, including environmental and genetic factors (Sher 2011). Genetic studies, such as family, twin and adoption studies, have consistently demonstrated that genetic factors appear to be involved in suicidal behavior (Wender et al. 1986; Roy et al. 1997; Li et al. 2010). These studies also illustrate that the predisposition to suicidal behavior is partly dependent on the presence of psychiatric disorders, such as bipolar disorder, schizophrenia, alcoholism and major depression disorder (MDD). Among these diseases, MDD is the most important predicting factor of suicidal behavior and eventually about 10 % of MDD patients may end up taking their lives by committing suicide (Winokur and Tsuang 1975). In addition, a large body of evidence indicates that various neural abnormalities, such as the dysfunction of glutamate receptor signaling and the reduced number and abnormal morphology of dendritic spines, are involved in the pathogenesis of many different brain diseases and suicidal behavior (Giuffrida et al. 2005; Govek et al. 2004; Szumlinksi et al. 2005).

Homer homolog 1 gene (*HOMER1*) is expressed pronouncedly in nervous system (Su et al. 2004). Previous studies reported that the encoded HOMER1 protein was involved in glutamatergic synapses and spine morphogenesis (Naisbitt et al. 1999; Tu et al. 1998; Hayashi et al. 2009; Govek et al. 2004), which suggested that the *HOMER1* gene may be an important candidate gene in the etiology of MDD and suicidal behavior. HOMER1a is a short isoform of HOMER1 and has a low expression level under normal conditions, but its expression level increases significantly after receiving neuronal activation (Brakeman et al. 1997). HOMER1b and 1c, the long isoforms of HOMER1, are constitutively expressed in vivo and in vitro without any activation (Kato et al. 1998). Both of the short and long isoforms share a conserved amino-terminal Enabled/vasodilator-stimulated phosphoprotein homolog 1 (EVH1) domain. This domain has a strong binding affinity to a proline-rich sequence, which can be found in Group 1 metabotropic glutamate receptors, NMDA glutamate receptor and scaffolding protein SHANK (Tu et al. 1998; Naisbitt et al. 1999; Hayashi et al. 2009). Moreover, Hayashi et al. demonstrated that HOMER1 and SHANK together formed a mesh-like matrix structure, which could serve as an assembly platform for other postsynaptic density (PSD) protein, such as mGluR1α/5, NMDA receptor and IP3 receptor (Hayashi et al. 2009; Shiraishi-Yamaguchi and Furuichi 2007). In addition, both long and short isoforms of HOMER1 protein could regulate cell-surface targeting and clustering of mGluR1α/5 (Roche et al. 1999; Ango et al. 2002; Serge et al. 2002). SHANK protein is an adaptor for the NMDA receptor/PSD-95 complex (Shiraishi-Yamaguchi and Furuichi 2007). Thus, we believe that HOMER1 protein has the ability to interact directly with mGluR1α/5 and indirectly with NMDA receptors at glutamatergic synapses. Moreover, the HOMER1 variants are involved in regulating spine morphogenesis, which is closely correlated with learning and memory performance (Govek et al. 2004). In addition, chronic stress could down-regulate the expression of HOMER1 in the prefrontal cortex of rat, and the effect could be reversed with antidepressant administration (Orsetti et al. 2008, 2009). Moreover, *HOMER1* knockout mice showed some changes that were similar to those observed in animal stress paradigms (Szumlinksi et al. 2005; Schmidt and Duman 2007), and these symptomatic phenotypes could be extinguished by overexpression of HOMER1 protein (Lominac et al. 2005).

Although there are several lines of evidence supporting the association of *HOMER1* polymorphisms with psychiatric disorders or suicidal behavior in Caucasians, the results may be significantly different in other ethnic populations due to the widely reported genetic heterogeneity (Wang et al. 2014; Yin et al. 2015; Thean et al. 2012). In humans, *HOMER1* is localized to chromosome 5q14.2, and several linkage studies demonstrated that susceptibility loci on this chromosome were significantly associated with schizoaffective disorder (Levinson et al. 2000; Gurling and Brynjolfsson 2001). Furthermore, Rietschel et al. found that two polymorphisms were nominally significantly associated with MDD by a genome-wide association study (Rietschel et al. 2010). One of the two polymorphisms is located in a putative regulatory region of *HOMER1* and had significant association with prefrontal activity during executive cognition. Additionally, *HOMER1* rs2290639 has been found to be associated with baseline psychopathology and antipsychotic treatment response in schizophrenic patients (Spellmann et al. 2011). Moreover, Strauss et al. performed a case–control association study in northern America and obtained a significant genotypic association between the rs2290639 and suicide attempts (Strauss et al. 2012).

In addition, broader personality profiles, impulsiveness and depression had long been linked with suicidal behavior and MDD (Duberstein 1995; Roy 2001; Corruble et al. 1999; Goldney et al. 2002; Horesh et al. 1997). The broader personality profiles include five-factor models: openness to experience (O), conscientiousness (C), extraversion (E), agreeableness (A), and neuroticism (N). Previous studies found that all the five factors were linked with suicidal behavior (Duberstein 1995; Roy 2001). Besides, it was believed that impulsive or depressed subjects had a higher chance of suicide attempts (Corruble et al. 1999; Horesh et al. 1997).

In this study, we aimed to investigate whether the *HOMER1* rs2290639 was associated with SA in Hong Kong Chinese since the previous association studies of the rs2290639 with SA were performed in Caucasians and the genetic heterogeneity exists between Caucasians and Chinese. The dbSNP database (release 142) shows that the allele frequencies of rs2290639 in Chinese Han Beijing (CHB) are 0.427 for A allele and 0.573 for T allele, but in Utah residents with Northern and Western European ancestry (CEU), they are 0.596 for A allele and 0.404 for T allele. Moreover, we also explored if the polymorphism was associated with MDD in Hong Kong Chinese. Besides, the potentially functional role of rs2290639 was briefly elucidated in this study. Another aim of this study was to find out whether there was any significant difference in psychometric properties among different groups of SA, non-SA, MDD and HC subjects.

## Methods

### Participants

Subjects who had their visit to either in-patient psychiatric unit of Shatin Hospital (Shatin, New Territories, Hong Kong) or out-patient psychiatric clinic of Prince of Wales Hospital (New Territories, Hong Kong) were recruited into this study. An interview was conducted for the subjects by experienced clinical psychiatrists, who acquired the subjects’ demographics, including sex, age, whether they had full-time job or study and a history of suicide attempts (SA). Besides, The Chinese-Bilingual Structured Clinical Interview for the Diagnostic and Statistical Manual of Mental Disorders, 4th Edition (Axis I, Patient version) (CB-SCID-I/P) was also carried out for the subjects to ascertain their depression status as it has a high prevalence in SA patients (Segal et al. 1994). Those subjects who suffered from mental retardation or dementia were excluded from this study. Finally, 117 SA patients (34 men and 83 women) and 167 MDD patients (44 men and 123 women) were included into this study. The mean age of SA patients was 38.5 ± 11.8 and that of MDD patients was 41.3 ± 11.9. Besides, those subjects suffering from any kind of psychiatric disorder but not having a history of SA would be considered as non-suicide attempters (non-SA), and those not suffering from any kind of psychiatric disorder and also not having a history of SA would be grouped into healthy controls (HC). 198 non-SA subjects [87 men and 111 women, mean age = 39.6 (±11.9) years] and 84 healthy controls [42 men and 42 women, mean age = 39.3 (±12.2) years] were recruited into this study. Moreover, the detailed information of those subjects was reported in Table 1.

**Table 1:** Information of SA, non-SA, MDD and non-MDD

Phenotypes/No. of subjects	MDD	Non-MDD	Sub-total
SA	72	45	117
Non-SA	95	103	198
Sub-total	167	148	315

*SA* suicide attempts, *non*-*SA* non-suicide attempts, *MDD* major depression disorder, *non*-*MDD* subjects without major depression disorder

As shown in Table 1, there were 72 patients that suffered from a comorbid history of MDD and SA. In addition, there were 45 non-MDD SA patients, most of whom had other mental disorders, such as bipolar disorder, schizophrenia and other mental disorders. However, 8 of them did not suffer from any mental disorders. Furthermore, all the 103 non-SA and non-MDD subjects had other mental disorders. Given the number of subjects with either bipolar disorder or schizophrenia was very small (<82), we did not perform association studies of rs2290639 with the two disorders. But the two disorders were considered as covariates when comparing SA group with non-SA group in rs2290639.

All the participants are Han Chinese in origin. Written informed consents were obtained from all the subjects. This study was reviewed and approved by the Joint Chinese University of Hong Kong—New Territories East Cluster Clinical Research Ethics Committee (CRE Reference No. 2006.393).

### Psychometric properties of subjects

All patients completed three clinical scales including NEO Personality Five-factor Inventory, Barratt Impulsiveness Scale (BIS) and Hospital Anxiety and Depression Scale (HADS). The NEO Personality Five-factor Inventory is a simplified version of NEO Personality Inventory Revised (NEO PI-R). It scores individuals on five personality dimensions: openness to experience (O), conscientiousness (C), extroversion (E), agreeableness (A) and neuroticism (N). A Chinese translated version of the inventory has been validated and it was supported by spouse rating of one’s personality. The test–retest reliability was found to be high (Yang et al. 1999). Moreover, the inventory was found to have high internal consistency in assessing personality traits (Eggert et al. 2007). BIS is a self-report measurement of impulsiveness. Translation (translated from English to Chinese) and back translation (back translated into English) has been performed in a study to assess the accuracy of translation (Yao et al. 2007; Patton et al. 1995). HADS is a self-rated scale which consisted of anxiety and depression subscales. The Chinese version has been translated and demonstrated good agreement with the English version (Leung et al. 1993, 1999). It is found to be reliable in measuring the severity of emotional disorder, especially for MDD (Bjelland et al. 2002).

### SNPs genotyping

Genomic DNA was extracted from two milliliters of saliva reagent using the Oragene™ DNA self-collection kit according to the manufacturer’s instruction (DNA Genotek, Inc., Ottawa, Canada). After DNA extraction, the concentration of genomic DNA was determined by Nanodrop 2000c spectrophotometer (Thermo Fisher Scientific Inc., MA, US). For PCR reactions, a 10 µl volume of PCR mixture containing 100 ng of genomic DNA was used for standard ABI TaqMan^®^ SNP Genotyping assays. The mixture was firstly treated by activation of the uracil-*N*-glycosylase for 2 min at 50 °C, followed by denaturation for 10 min at 95 °C and amplification over 45 cycles of 15 s at 95 °C and 1 min at 60 °C using ‘Genotyping’ option with three steps (pre-read, amplification and post-read) in ABI ViiA 7 Real-Time PCR system (Applied Biosystems, Foster City, California, USA).

### Bioinformatics prediction of transcription factors binding sites for *HOMER1* rs2290639

To investigate the possible molecular mechanism of the rs2290639, we performed the prediction of transcription factors binding sites for this polymorphism using four bioinformatics tools: P-Match v1.0 (TRANSFAC v6.0, Group of Matrices: vertebrates, Profiles: nerve-system-specific), PROMO v3.0.2 (TRANSFAC v8.3), JASPAR (JASPAR CORE Vertebrata) and AliBaba 2.1 (TRANSFAC v4.0) (Messeguer et al. 2002; Mathelier et al. 2014; Cartharius et al. 2005). The transcription factor found by more than one tool was considered as a potentially reliable binding factor.

### Statistical analyses

Comparisons of continuous and categorical variables were accomplished by t test and Chi square test respectively using SPSS program (version 20.0). Fisher’s exact test would be adopted when low cell counts or expected frequencies occurred in the analyses. Comparisons of more than two groups with continuous data would be performed by one-way ANOVA followed by Post Hoc Multiple Comparisons (Bonferroni). Comparisons of more than two groups with categorical data would be carried out by Chi square test followed by Mann–Whitney test. The correlations between impulsiveness and NEO personality five factors in SA and MDD patients were performed using bivariate correlation analysis. Hardy–Weinberg equilibrium (HWE) tests were used to examine the fit of the genotypic distributions of rs2290639 to equilibrium in each sample. The genotype and allele frequencies were compared by Chi square test or Fisher’s exact test. The odds ratio (OR) values and 95 % confidence intervals (CI) were determined by logistic regression analysis after adjustment of confounding factors. The different models’ power were calculated separately in Power and Sample Size Program (Dupont and Plummer 1990). Meta-analysis was conducted in StateSE (version 12.0, StataCorp LP, Texas, USA) (Boston and Sumner 2003). I^2^ statistics were adopted for the assessment of heterogeneity between different studies (Higgins and Thompson 2004; Trikalinos et al. 2008). Based on I^2^ values, different effect models, fixed effect model or random effect model, were adopted to meta-analysis. P values (2-tailed) of <0.05 were considered statistically significant.

## Results

### Demographics and psychometric properties of SA, non-SA, MDD and HC

**Fig. 1:**
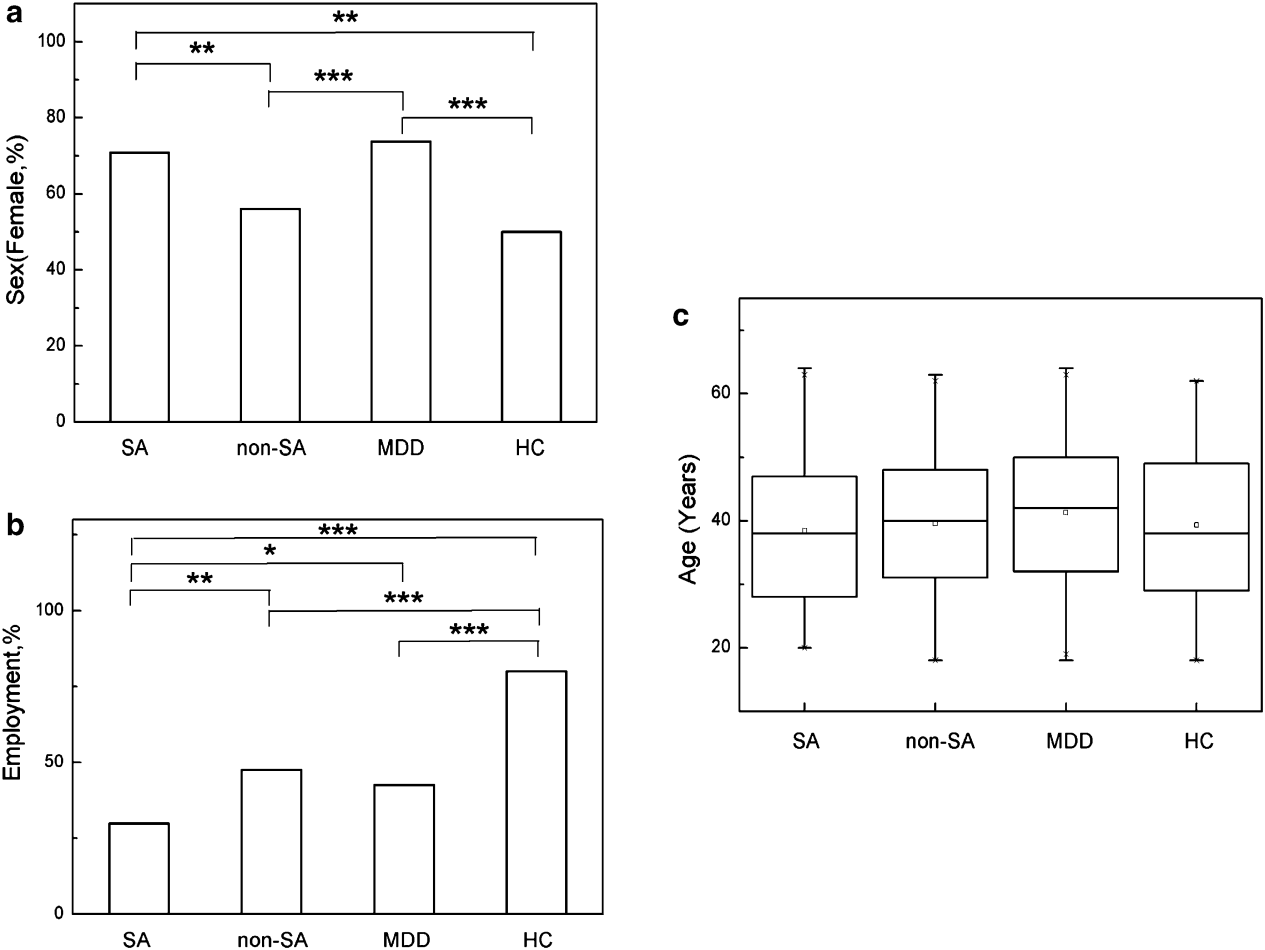
Demographics of SA, non-SA, MDD and HC (**a** sex, **b** employment, **c** age). *SA* suicide attempts patients, *non-SA* non-suicide attempts patients with psychiatric disorders, *MDD* major depressive disorder patients, *HC* healthy controls; data are presented in Median (Q1 and Q3) for age and in percentage for sex and employment

As shown in Fig. 1 and Additional file 1: Table S1, the percentage of female in SA and MDD patients was significantly higher than that in HC (P value = 0.003 and <0.001 respectively). Besides, there was also a significant difference between SA and non-SA patients (P value = 0.009). For the status of employment, SA patients had a significantly lower percentage of employment than the other three groups (P value = 0.002, 0.031 and <0.001 for non-SA, MDD and HC respectively). Furthermore, HC had a significantly higher percentage of employment than non-SA and MDD patients (P value <0.001 and <0.001 respectively). However, there was no significant difference of age in the four groups (P value >0.299).

**Fig. 2:**
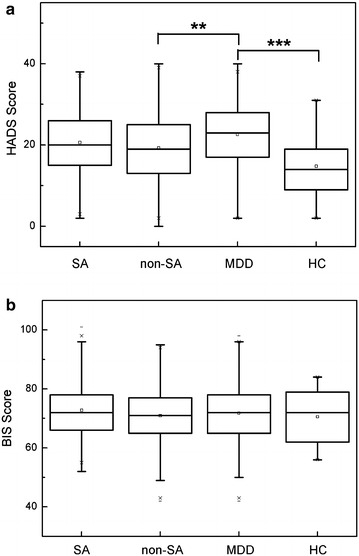
Psychometric properties of SA, non-SA, MDD and HC (**a** HADS score, **b** BIS score). *SA* suicide attempt patients, *non-SA* non-suicide attempts subjects with psychiatric disorders, *MDD* major depressive disorder patients, *HC* healthy controls; data are presented in Median (Q1 and Q3)

For the psychometric properties of subjects, MDD patients scored significantly higher in HADS score compared to non-SA patients and HC (P value = 0.001 and <0.001, respectively, Fig. 2 and Additional file 1: Table S1). Besides, the relationships between impulsiveness and NEO personality five factors in SA and MDD patients were to be investigated. In suicide attempters, impulsiveness was inversely correlated with NEO-conscientiousness (r = −0.55, P < 0.001), NEO-extroversion (r = −0.38, P < 0.001), NEO-agreeableness (r = −0.46, P < 0.001), but positively correlated with NEO-neuroticism (r = 0.41, P < 0.001). In MDD patients, impulsiveness had significantly inverse correlations with NEO-openness to experience (r = −0.31, P < 0.001), NEO-conscientiousness (r = −0.55, P < 0.001), NEO-extroversion (r = −0.28, P = 0.001) and NEO-agreeableness (r = −0.37, P < 0.001), but had positive correlation with NEO-neuroticism (r = 0.40, P < 0.001). However, we did not find that there was any significant difference for BIS score and NEO personality five factors in the four groups (Fig. 2 and Additional file 1: Table S1, data not shown for NEO personality five factors).

### Association study of *HOMER1* rs2290639 with suicide attempts and major depression disorder

The number of successfully genotyped DNA samples in SA, non-SA, MDD and HC were 111, 189, 160 and 84 respectively. The distribution of rs2290639 in HC was in accordance with the Hardy–Weinberg equilibrium (P value = 0.236). For the association study, rs2290639 was found to be significantly associated with suicide attempts in homozygous model (P value = 0.033, Table 2), and it remained significant in logistic regression analysis after controlling for covariates and after 2000 permutation tests (P value = 0.032 and 0.043, respectively). Moreover, the results in genotypic model also implied that this polymorphism was significantly associated with suicide attempts (adjusted P value after permutation tests is equal to 0.008). In the recessive model, SA patients had a significantly higher frequency of AA homozygote than non-SA subjects (P value = 0.004), and the difference was still significant in logistic regression analysis and after permutations tests (P value = 0.005 and 0.005, respectively. Table 2). The results of logistic regression analysis also indicated that the subjects with AA homozygote had 2.78-fold higher odds of SA than those with T-carrier genotypes (OR 2.78; 95 % CI 1.35–5.71). Following this finding, we further investigated whether there was any difference of psychometric properties between SA patients with homozygote (AA) and those with T-carrier genotypes. However, we did not find any difference in BIS score, HADS score or NEO personality five factors (Additional file 1: Fig. S1). Regarding to the SA (n = 72) and non-SA (n = 95) in subjects with MDD in this study (Table 1), the statistical analyses also demonstrated that there were significant associations of this polymorphism with SA (P value = 0.006 in Chi square test and adjusted P value = 0.007 in logistic regression analysis). Besides, no significant difference was found in genotypic frequency of rs2290639 between SA and HC as well as between MDD and HC (Table 2).

**Table 2:** Allele and genotype frequencies of *HOMER1* rs2290639 in HC, SA, MDD and non-SA

Model	HC (n = 84)	SA (n = 111)	MDD (n = 160)	Non-SA (n = 189)	SA versus HC		SA versus non-SA			MDD versus HC	
					P (*X* ^2^)	P (logistic)	P (*X* ^2^)	P (logistic)	P (Perm)	P (*X* ^2^)	P (logistic)
*Allelic*											
A	65 (38.7)	92 (41.4)	122 (38.1)	138 (36.5)	0.585	0.994	0.230	0.195	–	0.903	0.621
T	103 (61.3)	130 (58.6)	198 (61.9)	240 (63.5)							
*Genotypic*											
AA	10 (11.9)	22 (19.8)	23 (14.4)	16 (8.5)	0.226	0.833	*0.009*	*0.013*	*0.008*	0.653	0.606
AT	45 (53.6)	48 (43.3)	76 (47.5)	106 (56.1)							
TT	29 (34.5)	41 (36.9)	61 (38.1)	67 (35.4)							
*Homozygous*											
AA	10 (11.9)	22 (19.8)	23 (14.4)	16 (8.5)	0.326	0.844	*0.033*	*0.032*	*0.043*	0.839	0.823
TT	29 (34.5)	41 (36.9)	61 (38.1)	67 (35.4)							
*Heterozygous*											
AT	45 (53.6)	48 (43.3)	76 (47.5)	106 (56.1)	0.377	0.308	0.253	0.262	–	0.455	0.388
TT	29 (34.5)	41 (36.9)	61 (38.1)	67 (45.4)							
*Recessive*											
AA	10 (11.9)	22 (19.8)	23 (14.4)	16 (8.5)	0.139	0.354	*0.004*	*0.005*	*0.005*	0.592	0.568
AT + TT	74 (88.1)	89 (80.2)	137 (85.6)	173 (91.5)							

*HC* healthy controls, *SA* suicide attempt patients, *MDD* major depressive disorder patients, *non-SA* non-suicide attempts subjects with psychiatric disorders, *OR* odds ratio, *CI* confidence interval. Data is shown in number (%); P(*X* ^*2*^): P value were calculated by Chi square test; P (logistic): P value calculated by logistic regression analysis after controlling by sex and age in SA versus HC and MDD versus HC; P value calculated by logistic regression analysis after controlling by sex, age, MDD, bipolar disorder and schizophrenia in SA versus non-SA; P (Perm): adjusted P value after 2000 permutations tests for the significant results in Chi square tests; P values below 0.05 are in italics

In addition, a meta-analysis of this polymorphism with SA was performed including this study and a previous association study (Strauss et al. 2012). In total, 222 SA patients and 294 non-SA subjects were included in this meta-analysis. Regarding to a moderate heterogeneity across the two studies, random effect model was adapted to the heterozygous and recessive meta-analyses as the model allows for heterogeneity across studies (Trikalinos et al. 2008). The allele-A with lower frequency in our sample was considered as the minor allele. In recessive analyses (AA vs. AT + TT) of rs2290639, there was a significant difference between SA and non-SA groups in Hong Kong Chinese sample [OR (95 % CI) 2.78 (1.35–5.71)] and in the overall test [OR (95 % CI) 2.01 (1.16–3.52)], but not in Strauss’ sample [OR (95 % CI) 1.57 (0.85–2.89)] (Table 3). Moreover, this polymorphism was also significantly associated with SA in homozygous model [OR (95 % CI) 1.86 (1.05–3.29)]. Although there was a significant association in Strauss’ sample in heterozygous model [OR (95 % CI) 0.31 (0.15–0.67)], this polymorphism was not significantly associated with SA in Hong Kong Chinese sample [OR (95 % CI) 0.74 (0.43–1.26)] and in over-all test [OR (95 % CI) 0.50 (0.21–1.16)].

**Table 3:** Meta-analysis of association study of *HOMER1* rs2290639 and suicide attempts in three different models

Models (rs2290639)	Hong Kong Chinese sample	Strauss’ sample	Overall test	
	OR (95 % CI)	OR (95 % CI)	OR^b^ 95 % CI	I^2^ (P)^a^
*Homozygous*				
AA versus TT*	*2.36 (1.08*–*5.19)*	1.43 (0.63–3.24)	*1.86 (1.05*–*3.29)*	0 % (0.39)
*Heterozygous*				
AT versus TT*	0.74 (0.43–1.26)	*0.31 (0.15*–*0.67)*	0.50 (0.21–1.16)	70.8 % (0.06)
*Recessive*				
AA versus AT + TT*	*2.78 (1.35*–*5.71)*	1.57 (0.85–2.89)	*2.01 (1.16*–*3.52)*	28.7 % (0.24)

The OR and 95 % CI in significant associations were in italics; the genotype with an asterisk acts as the reference

^a^I^2^ (P): heterogeneity test for meta-analysis (P value)

^b^Fixed effect model was adapted to homozygous meta-analysis and random effect model was employed to heterozygous and recessive meta-analyses

### Potentially functional role of *HOMER1* rs2290639

**Fig. 3:**
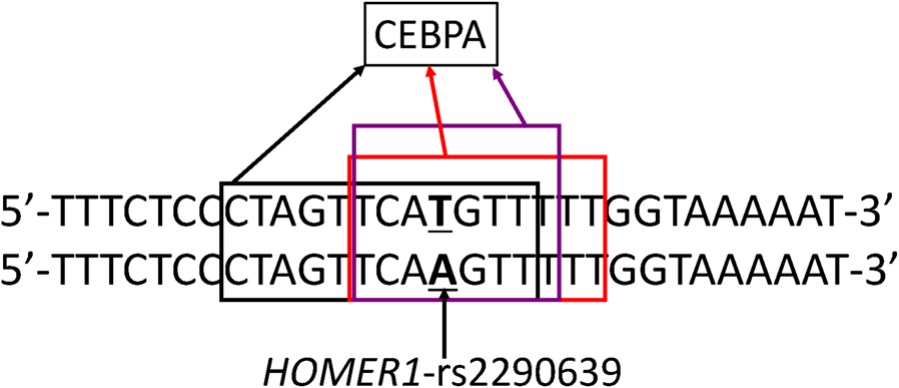
Bioinformatics prediction of transcription factors binding sites for *HOMER1* rs2290639. The predicted binding nucleotides by CEBPA in P-Match, Alibaba 2.1 and JASPAR are presented in *black*, *red* and *purple boxes* respectively

After prediction of potential binding regulatory elements by four bioinformatics tools, a transcription factor called C/EBP alpha (CEBPA) was found to be bound on rs2290639 and nearby nucleotides in three of the four tools (P-Match, Alibaba and JASPAR) (Fig. 3). Although there is a slight difference of bound nucleotides in the three tools, rs2290639 was covered by CEBPA in all the three tools. Miller et al. reported that CEBPA protein is a kind of bZIP transcription factor which can bind as a homodimer to certain enhancers (Miller et al. 2003). Moreover, previous studies reported that regulatory modules can be found in the promoter or exon regions of a gene, and it may also be located in the intron or downstream regions (Dong et al. 2010; Rosenthal et al. 1990; Pinaud et al. 1997; Pennacchio et al. 2013). Besides, the results of expression quantitative traits locus (eQTLs) demonstrated that rs2290639 was significantly associated with the expression level of the HOMER1 protein in thyroid tissue [P value = 5 × 10^−9^, the Genotype-Tissue Expression (GTEx) project, V6]. The results also showed that the subjects with AA homozygous genotype have a lower expression level of the HOMER1 protein compared with the subjects with T-carrier genotypes (effect size = −0.26). Thus, we propose that rs2290639 together with nearby nucleotides may act as a potentially regulatory module playing a functional role in the transcription of *HOMER1* gene, although the polymorphism was located on the downstream side of the *HOMER1* gene. In addition, we found that this polymorphism was located in the promoter region of an uncharacterized gene called *LOC101929201* in NCBI dbSNP database, and the distance between this polymorphism and the transcription start site of the gene is 635 bps. Therefore, rs2290639 may exert an important functional role through influencing the transcription of the *LOC101929201* gene.

## Discussion

This study suggested that *HOMER1* rs2290639 was significantly associated with SA in Hong Kong Chinese. Furthermore, the logistic regression analysis implied that rs2290639 AA homozygote was significantly associated with the susceptibility to SA in genotypic, homozygous and recessive models in Hong Kong Chinese sample (Table 2). And all the associations remained significant after 2000 permutation tests. Although Strauss et al. also found there was an association between the polymorphism and SA, the association was only found in heterozygous analysis according to the genotypic frequencies in Caucasians (Table 3). The different positively and negatively associated models in *HOMER1* rs2290639 observed in Chinese and Caucasians might be explained by the variations existing in human genetic structure, i.e. different ethnic populations show significant discrepancies in allelic or genotypic frequencies (Schork et al. 2001; Altshuler et al. 2005). Therefore, it was worthwhile to perform a meta-analysis including samples recruited in this study and Strauss’ study. According to the results of the meta-analysis, rs2290639 was significantly associated with SA in homozygous and recessive models (Table 3). Taken together, it is reasonable to conclude that *HOMER1* rs2290639 was significantly associated with susceptibility to SA in both Caucasians and Chinese affected by psychiatric disorders.

In addition, the potentially molecular mechanism behind the significant association was briefly elucidated in this study. *In silico* analysis in four tools for predicting transcription factors binding sites found that a reliable transcription factor called CEBPA binds on rs2290639 and nearby nucleotides in three of the four tools (Fig. 3). Moreover, the eQTLs results also implied that there was a strong association of this polymorphism and the expression level of the HOMER1 protein. Although rs2290639 is located on the downstream region of the *HOMER1* gene, this polymorphism together with nearby nucleotides may act as a regulatory module influencing the transcription of *HOMER1* as the regulatory module could be located in the intron region or downstream region (Fiskerstrand et al. 1999; Lovejoy et al. 2003; Baum et al. 2008; Rosenthal et al. 1990). Previous studies reported that one repeat length polymorphism (STin2), albeit located in the second intron of *SLC6A4* (serotonin transporter) gene, had functional role in the expression of SLC6A4 (Lovejoy et al. 2003; Fiskerstrand et al. 1999). Moreover, several polymorphisms or haplotypes in the first intron of *DGKH* (diacylglycerol kinase eta) gene were reported to be associated with bipolar disorder in Caucasians and Chinese (Baum et al. 2008; Zeng et al. 2011). Our group found that a different polymorphism, also located in the first intron of *DGKH* gene, was significantly associated with bipolar disorder in Hong Kong Chinese (Rao et al. 2015). Besides, Rosenthal et al. reported that an enhancer element was located downstream of the coding region in *MLC* (myosin light chain) 1/3 gene (Rosenthal et al. 1990). Thus, the regulatory function of rs2290639 and the interaction between rs2290639 and CEBPA are worthy to be investigated by further experimental approaches to determine how they influence the transcription of the *HOMER1* gene.

Taken together, it is reasonable to propose that the significant association between rs2290639 and SA may be explained by the polymorphism’s potentially functional role in regulating the transcription of the *HOMER1* gene, which acts a vital role in post-synaptic density and neurotransmission. HOMER1 protein is a key molecule at the post-synaptic membrane and constructs a polymeric network at post-synaptic density with SHANK3 (Hayashi et al. 2009), and this network could let HOMER1 be involved in regulating the function of postsynaptic receptors, such as serotonin receptor, dopamine D1 receptor, *N*-methyl-d-aspartate (NMDA) glutamate receptor and other receptors (Dell’aversano et al. 2009; Iasevoli et al. 2009). Those receptors related to serotonin, dopamine and glutamate were well-known to be associated with psychiatric disorders or suicidal behavior. The other proteins playing important roles in the polymeric network are worthy to be explored for their roles in the etiology of psychiatric disorders and suicidal behavior.

Besides, rs2290639 is located in the promoter region of an uncharacterized gene and might also exert its functional role through affecting the transcription of this gene. However, these hypotheses should be investigated by further experimental approaches. In addition, we did not find any difference in allelic frequencies neither or in any of the models between MDD and HC. It seems that rs2290639 is not associated with MDD in Hong Kong Chinese, although *HOMER1* might have a role in animal model under stress condition (Orsetti et al. 2008; Lominac et al. 2005). Furthermore, the negative results with MDD might be owing to the relatively moderate sample size of MDD and HC.

For the psychometric properties of subjects, we found that there was a close correlation between impulsiveness and NEO personality five factors in SA patients and MDD patients, which provides a possible way to assess the impulsiveness of patients through subjects’ personality profiles for Hong Kong Chinese as similarly reported in Caucasians (Miller et al. 2012a). Additionally, MDD patients had a significant higher score in HADS suggesting that HADS is also a reliable scale in measuring the depression status for Hong Kong Chinese. Moreover, the lower levels of employment in SA and MDD patients suggested that the worse psychological status may bring a reduced rate of employment for Hong Kong SA and MDD patients, which reconfirmed that these disorders would cause great suffering to their family and bring a heavy burden to the social healthcare system.

One limitation of this study was that lifetime history of suicide attempts relied on self-reports from the subjects, which may likely lead to recall bias. However, we attempted to overcome this limitation by semi-structured clinical interview with experienced clinical psychiatrists. Moreover, the relatively modest sample size might affect the validity of polymorphic association study. Although neither of the power in homozygous model nor in heterozygous model has a power above 80 %, the power in recessive model with a stronger effect size could achieve a power of 83.1 %. Nonetheless, the negative association of rs2290639 with MDD should be interpreted with caution. Besides, the meta-analysis performed in this study should also be interpreted with caution because in Strauss’ sample the values of odds ratio and confidence intervals determined in logistic regression analysis have not been adjusted for sex, age and Childhood-onset Mood Disorders.

In conclusion, this study suggested that the *HOMER1* rs2290639 was significantly associated with susceptibility to SA in Chinese affected by psychiatric disorders. This polymorphism might influence the transcription of the *HOMER1* gene as it changes the sequence of the transcription factor binding site. More related genes in post-synaptic density should be investigated to further understanding the etiology of psychiatric disorders and suicidal behavior.

## Additional file

10.1186/s40064-016-2404-1Demographics and psychometric properties of SA, non-SA, MDD and HC. **Figure S1.** Psychometric properties of SA patients with AA homozygote and SA patients with T-carrier genotypes.
