# Supplementary material for: Association of HOMER1 rs2290639 with suicide attempts in Hong Kong Chinese and the potentially functional role of this polymorphism
Source: Springerplus. 2016 Jun 17;5(1):767. doi: 10.1186/s40064-016-2404-1 (PMC4912501; doi:10.1186/s40064-016-2404-1)
Supplement: Supplementary file 1 — 10.1186/s40064-016-2404-1Demographics and psychometric properties of SA, non-SA, MDD and HC. Figure S1. Psychometric properties of SA patients with AA homozygote and SA patients with T-carrier genotypes. [file 40064_2016_2404_MOESM1_ESM.docx]

**S1 Table. Demographics and psychometric properties of SA, non-SA, MDD and HC.**

| Phenotype ^a^ | | SA | non-SA | MDD | HC |
| --- | --- | --- | --- | --- | --- |
| Number | | 117 | 198 | 167 | 84 |
| Sex  n (%) | Male | 34(29.1) | 87(43.9) | 44(26.3) | 42(50.0) |
|  | Female | 83(70.9) | 111(56.1) | 123(73.7) | 42(50.0) |
| Age  Mean ± SD | | 38.5±11.8 | 39.6±11.9 | 41.3±11.9 | 39.3±12.2 |
| Employment  n (%) | | 35(29.9) | 94(47.5) | 71(42.5) | 67(79.8) |
| BIS  Mean ± SD | | 72.8±10.0 | 71.0±9.4 | 71.8±9.6 | 70.6±10.7 |
| HADS  Mean ± SD | | 20.6±7.9 | 19.3±8.5 | 22.6±8.0 | 14.8±8.4 |

^a^ SA: suicide attempts; non-SA: non-suicide attempts patients with psychiatric disorders; MDD: major depressive disorder patients; HC: healthy controls; BIS: Barratt Impulsiveness Scale; HADS: Hospital Anxiety and Depression Scale.

**S1 Fig.** **Psychometric properties of SA patients with AA homozygote and SA patients with T-carrier genotypes.**

SA: suicide attempts; BIS: Barratt Impulsiveness Scale; HADS: Hospital Anxiety and Depression Scale; NEO-O: NEO-openness to experience; NEO-C: NEO-conscientiousness; NEO-E: NEO-extroversion; NEO-A: NEO-agreeableness; NEO-N: NEO-neuroticism.
